# Deep Learning Approaches for Multiple Sclerosis Detection in MRI Images

**DOI:** 10.1155/bmri/5726771

**Published:** 2026-04-06

**Authors:** Mohamad Marwan El Sidani, Rita Younes, Charles Yaacoub, Roy Abi Zeid Daou

**Affiliations:** ^1^ Faculty of Engineering, Université La Sagesse, Beirut, Lebanon; ^2^ ICL, Junia, Université Catholique de Lille, LITL, Lille, France

**Keywords:** CNN, DL, medical imaging, MRI, MS, ResNet, VGG16

## Abstract

Multiple sclerosis (MS) is a chronic neurological disorder commonly diagnosed through magnetic resonance imaging (MRI). Manual interpretation of MRI scans is time‐consuming and prone to observer variability. This study explores the use of deep learning (DL) models to automate MS lesion detection in MRI images. Four convolutional neural network (CNN) architectures—AlexNet, VGG16 pretrained on ImageNet, ResNet‐10, and DenseNet‐121—were utilized with slight modifications to optimize their performance for medical imaging tasks. The dataset, comprising 2831 FLAIR and T2 MRI slices from 60 patients, was extensively preprocessed, including normalization, resizing, and augmentation. The models were trained using the Adam optimizer and binary cross‐entropy loss function and evaluated on metrics such as accuracy, precision, recall, and *F*1‐score. VGG16 demonstrated the highest performance, with an area under the curve (AUC) of 0.94, followed by DenseNet‐121 with an AUC of 0.91, and both AlexNet‐C and ResNet‐10 with an AUC of 0.90. VGG16 also achieved the highest precision (0.81) and recall (0.93), resulting in the top *F*1‐score (0.86). ResNet‐10, however, showed the best balance between efficiency and performance, making it highly suitable for deployment in resource‐constrained environments, while DenseNet‐121 and AlexNet‐C were resource‐inefficient given their comparable performance to ResNet‐10. These findings underscore the potential of DL models to automate MS lesion detection, offering reliable, efficient, and scalable solutions for clinical diagnostics.

## 1. Introduction

Multiple sclerosis (MS) is a debilitating chronic disease affecting the central nervous system, leading to a wide range of neurological symptoms. It primarily manifests through the immune system′s attack on myelin, a fatty substance that insulates nerve fibers, leading to scarring or lesions on the brain, spinal cord, and optic nerves. Early and accurate diagnosis is crucial in managing the disease and improving patient outcomes, as timely treatment can potentially slow the progression and reduce neurological damage [1–5]. Left untreated, MS causes substantial disability in 80%–90% of individuals within 20–25 years of onset [6].

Over the years, magnetic resonance imaging (MRI) has emerged as an indispensable tool in the detection and monitoring of MS due to its ability to visualize the brain and spinal cord in detail. However, interpreting MRI scans is a complex task requiring a high level of expertise, often time‐consuming, and subject to interobserver variability [7–9]. These challenges have led to the exploration of advanced technologies to aid in diagnosis.

### 1.1. Related Work

The integration of machine learning (ML) and deep learning (DL) in medical diagnostics has shown promising potential in enhancing the accuracy of MS diagnosis. A significant contribution to this field is the comprehensive review by Aslam et al. [10]. This review evaluates various ML models used for diagnosing MS from 2011 to 2022, highlighting the prevalent use of support vector machines (SVMs), random forests (RFs), and convolutional neural networks (CNNs). It addresses challenges such as differentiating MS from other neurological conditions, ensuring patient data confidentiality, and the need for large, reliable datasets. The review also discusses opportunities for using secure data platforms, employing advanced AI solutions, and integrating diverse data types to enhance diagnostic accuracy. One significant finding was that SVMs achieved an accuracy of 85% in differentiating MS from other neurological conditions, while RF models showed improved performance with accuracies up to 87%. CNNs, particularly those with transfer learning, demonstrated even higher accuracies, with some models reaching 90%.

Building on this foundation, Shoeibi et al. provided a detailed analysis in their review [11]. This review focuses on the application of DL in MRI‐based diagnosis systems, examining various computer‐aided diagnosis systems (CADSs), essential preprocessing techniques, and the effectiveness of DL models compared to conventional ML. Shoeibi et al. reported that 2D‐CNN models achieved a dice similarity coefficient (DSC) of 66.55% on the ISBI 2015 dataset. They also noted that a 3D‐CNN model achieved a sensitivity of 80.21% and a specificity of 79.16% in predicting future disease activity. The review highlighted ongoing challenges such as data stratification, validation protocols, and the need for large datasets, alongside the evolution of diagnostic applications, MRI modalities, and DL architectures.

Moreover, DL, particularly through CNNs, has significantly advanced MS diagnosis. CNNs have demonstrated remarkable capabilities in analyzing medical images like MRI scans to identify subtle and highly variable MS‐related structures. The comprehensive survey by Sahu and Dash [12] categorizes DL methods into standalone CNNs, hybrid models that integrate CNNs with traditional classifiers, and deep transfer learning models. These models are meticulously analyzed for their architecture, preprocessing steps, feature extraction techniques, and overall accuracy in MS classification. They reported that a 14‐layer CNN with batch normalization and stochastic pooling achieved an accuracy of 98.77%, a sensitivity of 98.77%, and a specificity of 98.76% on a dataset of 38 MS patients. Another model, a hybrid CNN‐RNN, demonstrated an accuracy of 95.2% and an area under the curve (AUC) of 0.97, highlighting the potential of combining spatial and temporal features for improved diagnosis.

Afzal et al. [13] utilized a 2D‐CNN model trained on the John Hunter Hospital′s dataset, achieving an accuracy of 100%. Roy et al. [14] used a 2D‐CNN model for lesion segmentation on the ISBI 2015 dataset, achieving a DSC of 52.4%. Alijamaat et al. [15] demonstrated a 2D‐CNN model on the eHealth Laboratory dataset, achieving an accuracy of 99.05%, a sensitivity of 99.14%, and a specificity of 98.89%.

However, despite these advancements, several challenges persist. Many existing studies tend to focus on specific types of CNN architectures without fully exploring the comparative performance of a broader range of models, from simple to complex. This narrow focus limits the understanding of how different architectural choices impact diagnostic accuracy and computational efficiency in the context of MS lesion detection. Additionally, the consideration of model complexity—such as computational requirements and model size—has often been overlooked, yet it is a critical factor for the practical deployment of these models in clinical settings. Furthermore, reproducibility remains a significant challenge in this domain. Variability in datasets, lack of standardization in model training, and inconsistent reporting of methodologies have made it difficult for researchers to replicate findings accurately, which hampers the progress of developing reliable AI tools for clinical use. There is a need for comprehensive studies that not only compare various CNN architectures but also evaluate the trade‐offs between their complexity and performance while ensuring that the research can be reliably reproduced and applied in real‐world settings.

### 1.2. Contributions

This study presents several significant contributions to the application of CNNs for MS lesion detection using MRI images. The key contributions are as follows:

1. *Comprehensive evaluation of CNN architectures*: We conducted an extensive evaluation of a range of CNN architectures, from the relatively simple ResNet‐10 [16] to the more complex DenseNet‐121 [17], as well as the classic AlexNet [18] and VGG16 [19] models. This evaluation includes the use of transfer learning, particularly with VGG16, which leverages pretrained weights from large‐scale image datasets to enhance model performance in the specific task of MS lesion detection. Although these models represent early architectures primarily used as backbones in more recent frameworks, enhancing their performance could benefit both legacy systems and modern architectures where they serve as foundational elements.
2. *In-depth comparative analysis including model complexity*: The study provides a detailed comparative analysis of these CNN models, not only in terms of standard performance metrics such as accuracy, precision, recall, and AUC but also by incorporating model complexity as a key factor. This includes evaluating the trade‐offs between the complexity of the models—roughly measured as the model size—and their diagnostic performance, to identify the most suitable architecture for clinical applications.
3. *Identification of optimal architectures for practical use*: The study identifies CNN architectures that are particularly well suited for MS lesion detection in clinical settings. The results highlight the effectiveness of transfer learning in complex models like VGG16 and DenseNet‐121 while also demonstrating that simpler architectures like ResNet‐10 can achieve competitive performance with significantly reduced complexity. This balance between accuracy and efficiency is crucial for the deployment of AI tools in real‐world medical environments.

## 2. Materials and Methods

### 2.1. Data Description

The dataset utilized in this study is sourced from [20]. The dataset comprises multisequence MRI data of 60 MS patients, complete with consensus manual lesion segmentation, Expanded Disability Status Scale (EDSS) scores, and comprehensive patient information. The EDSS is a method of quantifying disability in MS and monitoring changes in the level of disability over time. The scale ranges from 0 to 10 in 0.5 increments, with higher scores indicating greater disability. The segmentation images are binary masks, where white pixels indicate the presence of MS lesions, aiding in precise and automated lesion detection. This segmentation was meticulously validated by three radiologists and a neurologist, ensuring high‐quality, reliable data for our research.

The detailed patient information and the precise manual lesion segmentation, along with their validation by multiple experts, provide a strong foundation for developing robust and reliable diagnostic models.

At the data level, both T2 and FLAIR MRI images play essential roles in identifying MS lesions, with T2 highlighting areas of inflammation and demyelination and FLAIR providing better lesion contrast, especially near ventricles and in the brain′s white matter. In MS diagnosis and monitoring, the presence, size, and distribution of these lesions across different brain regions help assess disease progression.

### 2.2. Data Compilation and Labeling

**Figure 1:**
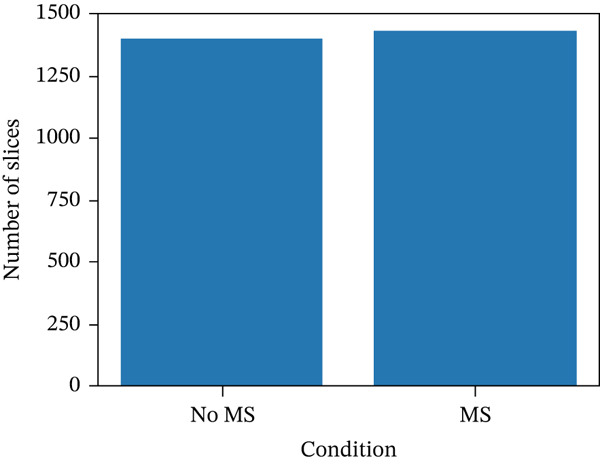
Distribution of MS and non‐MS classes in the dataset.

The dataset includes 2831 slices from 60 patients when taking both T2 and Flair into account. Binary labels were assigned based on the presence of MS lesions, identified through the presence of white pixels in the corresponding binary masks. Binary labels were assigned at the slice level based on the manual lesion masks: A slice was labeled as lesion‐positive (Label 1) if its corresponding mask contained lesion pixels and lesion‐negative (Label 0) otherwise. For readability, we refer to lesion‐positive slices as “MS” slices and lesion‐negative slices as “non‐MS” slices. Importantly, the resulting slice‐wise dataset is approximately balanced, with the number of lesion‐positive slices being close to the number of lesion‐negative slices (Figure 1). Consequently, no additional class‐balancing techniques were required for this slice‐wise classification task. This contrasts with pixel‐wise lesion segmentation, where the lesion class is typically highly underrepresented relative to the background.

### 2.3. Data Preprocessing

**Figure 2:**
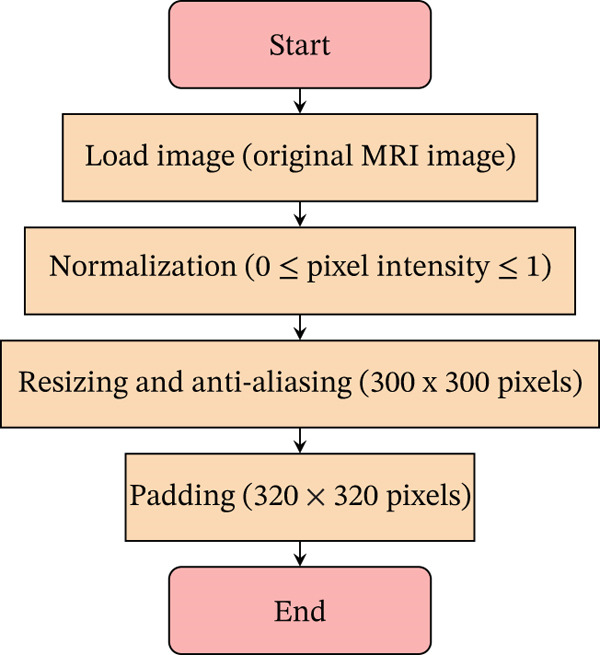
Flowchart of data preprocessing steps. Each step includes specific operations to standardize the MRI images for neural network training.

Data preprocessing was conducted using Python in a Google Colab environment, where the dataset was mounted from Google Drive. Each image underwent a series of operations to standardize the input for neural network training, as shown in Figure 2:

• *Scaling*: Each image was scaled so that pixel values fall within the range of 0–1. This step is essential for maintaining numerical stability and ensuring that all input features contribute uniformly during the learning process.
• *Padding*: All images were padded directly to maintain the 1:1 ratio.
• *Resizing*: After padding, images were resized to 320 × 320.

**Figure 3:**
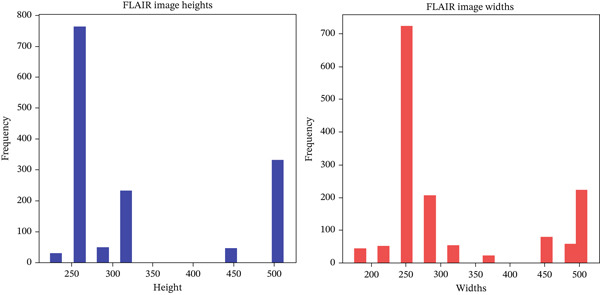
Distribution of FLAIR Image heights and widths.

**Figure 4:**
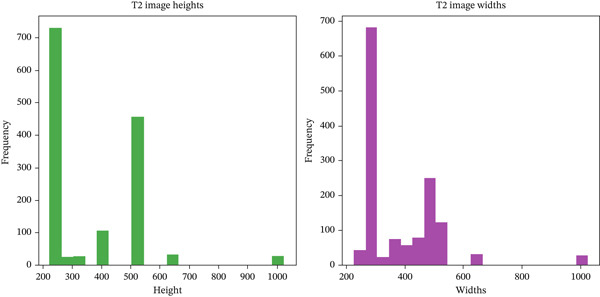
Distribution of T2 Image heights and widths.

The choice of 320 × 320 as the final image size balances computational efficiency with the need to preserve detail. This dimension is practical, minimizing resizing or padding and thus maintaining the integrity of the original images. It is compatible with various CNN architectures and optimizes the use of computational resources, facilitating effective feature learning and model performance. The decision was made after plotting image size distributions, as shown in Figures 3 and 4.

### 2.4. Dataset Split

The dataset was split into 80% for training and validation and 20% for testing, with a further 25% of the training set allocated for validation to fine‐tune models. This strategic split ensures a comprehensive training process while retaining a substantial portion of the data for unbiased evaluation of the model′s performance.

### 2.5. Data Augmentation

To enhance model robustness and introduce variability that mimics clinical settings, data augmentation techniques were applied during training. These augmentations included the following:

• *Random intensity adjustments*: The intensity of images was randomly scaled by a factor between 0.9 and 1.1 and shifted by a value within the range of −0.05 to +0.05. After these adjustments, pixel values were clipped to ensure they remained within the normalized range of 0–1. These adjustments simulate minor variations in scan brightness and contrast, reflecting different MRI scanner settings, while maintaining the integrity of the image data.
• *Geometric transformations*: Images underwent random rotations, translations, and flips. These transformations help the model learn to recognize pathological features from various orientations and positions, increasing its diagnostic accuracy on unseen data.
• *Noise injection*: Gaussian noise was added to the images to simulate the effect of MRI scanner noise and improve the model′s tolerance to such imperfections. Gaussian noise was chosen for its mathematical simplicity and ability to generalize across different types of noise, making the model more robust to various noise conditions commonly encountered in medical imaging.

These augmentation strategies were implemented using Keras′s *ImageDataGenerator*, allowing for on‐the‐fly image manipulation during model training, ensuring no two images seen by the model are exactly the same. This approach not only prevents overfitting but also prepares the model for the variability encountered in real‐world clinical environments.

The preprocessing and augmentation setup is designed to ensure that all portions of the image, including edges potentially containing lesions, are effectively used for training the model, thus maximizing the clinical relevance of the model predictions.

## 3. Model Architectures

We implemented four different CNN architectures, incorporating minor adjustments to optimize their performance for MS lesion detection in MRI images. These adjustments were made to leverage the inherent strengths of each architecture while ensuring they were well suited to the specific characteristics of our dataset.

### 3.1. Customized AlexNet Architecture

**Figure 5:**
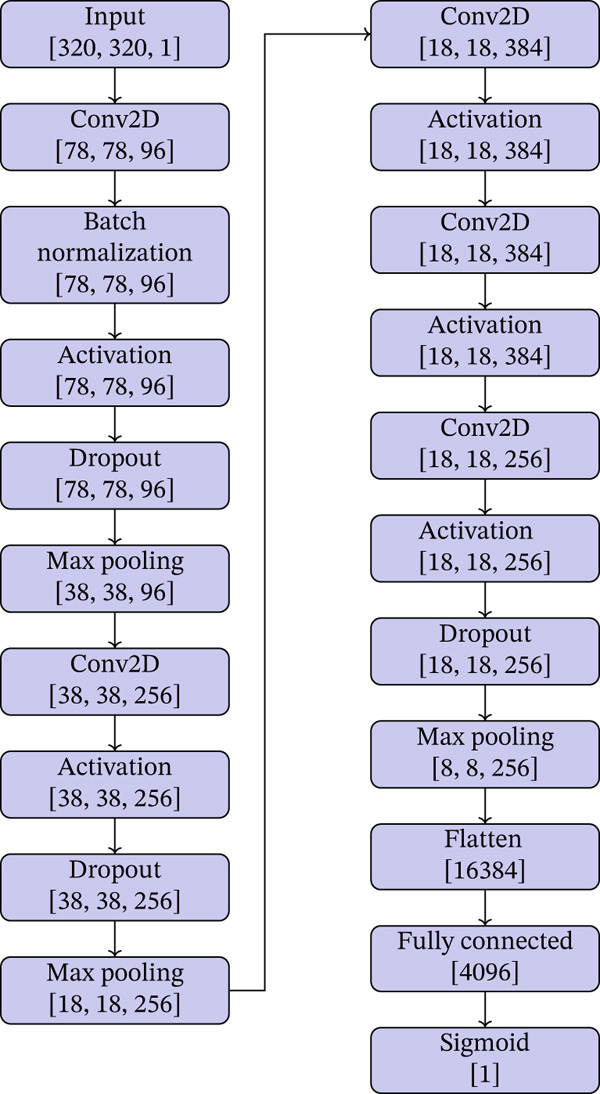
AexNet‐C architecture for MS detection.

Our adaptation of the AlexNet architecture incorporates additional regularization and dropout layers to prevent overfitting, which is crucial given the high variability within MRI data related to MS. Detailed empirical analysis was conducted to determine the optimal placement and probability of dropout layers, as well as the integration of batch normalization, to achieve a balanced model that avoids underfitting while managing the high capacity of the network to generalize over unseen data. The customized AlexNet model (AlexNet‐C) used in our studies is depicted in Figure 5 and is summarized as follows:

• *Input layer*: Accepts images of size 320 × 320 pixels, adjusting the original AlexNet input to accommodate our preprocessed MRI data.
• *First convolutional layer*: Features a large 11 × 11 kernel at a stride of 4, quickly reducing the spatial dimensions, followed by batch normalization, ReLU activation, and dropout with a rate of 25%.
• *Second convolutional layer*: Utilizes a 5 × 5 kernel to further detail feature extraction, followed by ReLU activation, and another dropout layer at 25%. A max pooling layer reduces spatial dimensions further.
• *Third to fifth convolutional layers*: Each uses 3 × 3 kernels, with each layer followed by ReLU activation. The fifth layer includes a dropout at 25% before proceeding to a max pooling layer.
• *Max pooling layers*: Placed to reduce dimensionality at specific points in the network, which helps in making the representation smaller and more manageable.
• *Flattening layer*: Converts the multidimensional input into a flat array that feeds into the fully connected layers.
• *Fully connected layers*: The network includes two dense layers leading up to the final output layer. The first dense layer has 4096 units, followed by a second dense layer functioning as the output layer with a single neuron using a sigmoid activation function for binary classification (presence of MS lesions).
• *Regularization*: L2 regularization is applied to all convolutional and fully connected layers to penalize large weights, aiding in the model′s generalization capabilities.

This architecture is designed to robustly handle the complexities of MRI data by focusing on high‐level features in the initial layers and more detailed features in the deeper layers. The modifications, particularly the increased dropout and regularization, are tailored to address the overfitting challenge common in medical imaging tasks where the dataset size is relatively small compared to mainstream image classification tasks.

### 3.2. VGG16 Architecture

**Figure 6:**
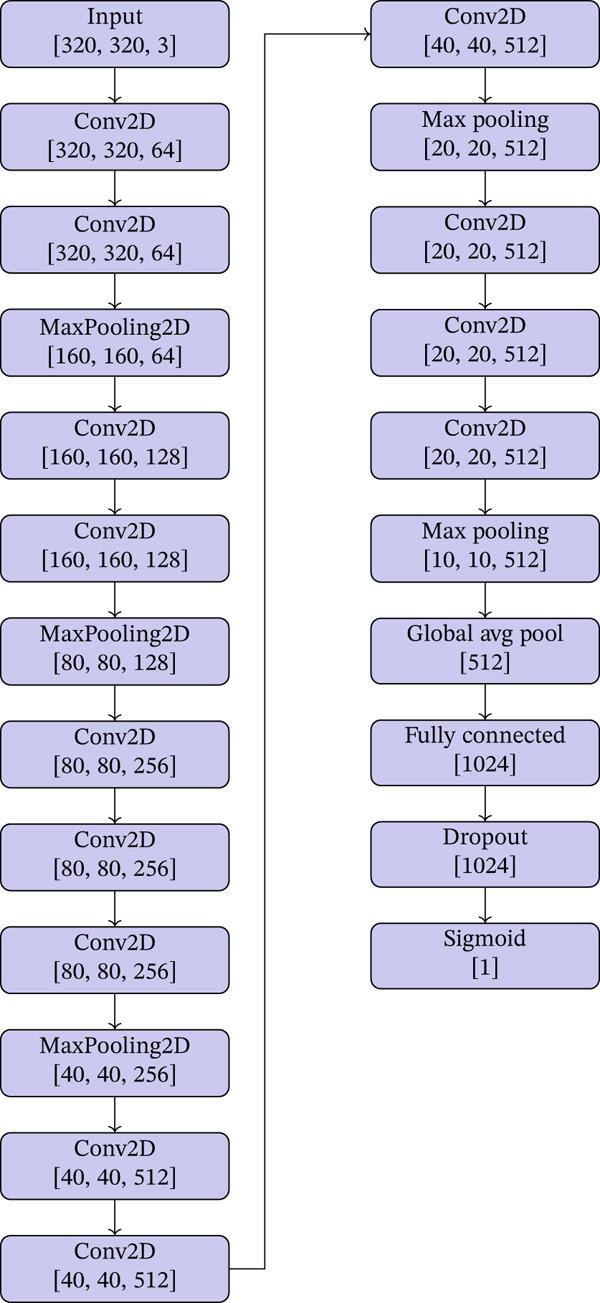
VGG16 architecture for MS detection.

We utilized theVGG16 architecture, originally developed for ImageNet classification, to perform binary classification for detecting MS lesions in MRI images. VGG16 was selected not only for its deep structure and capability to extract intricate features but also for the advantages it offers through transfer learning. By employing a VGG16 model pretrained on ImageNet, we were able to leverage the rich, prelearned feature representations from a vast and diverse dataset. This approach allows the model to quickly adapt to the specific task of MS lesion detection, enhancing performance and reducing the need for extensive labeled medical data. The specific VGG16 model architecture used in our study is illustrated in Figure 6 and is summarized as follows:

• *Base model*: We utilized the pretrained VGG16 model, with weights pretrained on ImageNet. This model′s architecture was modified to accept our input size of 320 × 320 pixels, reflecting the resolution enhancement from our preprocessing steps.
• *Layer freezing*: The layers up to the fourth block were frozen to utilize the generic features learned from ImageNet, while the last block′s layers were made trainable to adapt to the specifics of MRI images.
• *Convolutional layers*: VGG16′s numerous convolutional layers use small 3 × 3 kernels, which allow the network to learn a rich feature representation. The depth of the network captures complex structures in the imagery, crucial for identifying subtle anomalies indicative of MS.
• *Custom top layers*: Instead of the typical fully connected layers, we introduced a Global Average Pooling layer to reduce overfitting by minimizing the total number of trainable parameters.
• *Classification layers*: A dense layer with 1024 units follows, featuring ReLU activation, coupled with a 50% dropout rate.
• *Output layer*: The final layer is a dense layer with a sigmoid activation function designed for binary classification, indicating the presence or absence of MS lesions.
• *Regularization*: L2 regularization is applied to all trainable layers to enhance the model′s ability to generalize, preventing overfitting on the MRI dataset, which is relatively smaller and more specific compared to typical ImageNet‐scale datasets.

### 3.3. ResNet Architecture

ResNet architectures are well regarded for their ability to train deep neural networks by effectively addressing the vanishing gradient problem through the use of skip connections. These connections facilitate the flow of gradients throughout the network without significant loss, making ResNet particularly effective for the complex feature extraction needed in tasks like MS lesion detection from MRI scans.

The skip connections not only prevent vanishing gradients but also simplify the optimization process. As highlighted by [21], these connections help smooth the loss landscape, improving both the optimization behavior and the learning efficiency—critical attributes in medical imaging, where precise feature extraction is essential for accurate diagnosis.

**Figure 7:**
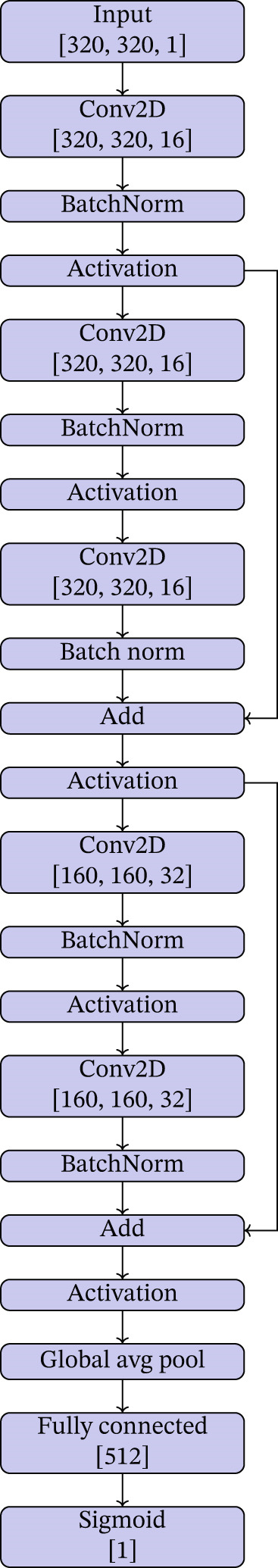
ResNet‐10 architecture for MS lesion detection in MRI.

Our model′s architecture, referred to as ResNet‐10 due to it having 10 convolutional layers, is depicted in a simplified form in Figure 7, which shows a version with two residual blocks. Below is a detailed breakdown of the architecture:

• *Input layer*: The model accepts grayscale MRI images of size 320 × 320 pixels, aligning with typical MRI resolution.
• *Initial convolutional layer*: The network begins with a 3 × 3 convolutional layer containing 16 filters, followed by batch normalization and ReLU activation. This layer is designed to effectively capture primary features from the input images.
• *Residual blocks*: The model includes four residual blocks, each composed of two 3 × 3 convolutional layers with increasing filter sizes (16, 32, 64, and 128). Each convolutional layer is followed by batch normalization and ReLU activation. These blocks allow the network to learn increasingly complex features as it deepens.
• *Skip connections*: Each residual block incorporates skip connections that add the input of the block directly to the output after the second convolutional layer. This integration ensures effective gradient flow through the network, stabilizing the training process, especially in deeper layers.
• *Global Average Pooling*: Following the residual blocks, a Global Average Pooling layer is applied to compress each feature map into a single value, reducing the number of parameters and helping to combat overfitting.
• *Fully connected layer*: Before the final output, the network includes a fully connected layer with 512 units and ReLU activation. This layer enhances the model′s capacity to learn complex patterns from the compressed feature maps.
• *Output layer*: The model concludes with a single neuron with a sigmoid activation function, specifically designed for binary classification, such as detecting the presence or absence of MS lesions.

### 3.4. DenseNet‐121 Architecture

DenseNet architectures are distinguished by their dense connectivity pattern, where each layer is connected to every other layer in a feed‐forward fashion. This unique setup facilitates substantial improvements in gradient flow, feature propagation, and reuse, making the network highly efficient and reducing the number of parameters. Dense connectivity also inherently incorporates the benefits of deep supervision, with each layer receiving additional supervision from the loss function through the shorter connections.

Moreover, the dense connections in DenseNet help in smoothing the loss landscape, similar to the effect seen in ResNet architectures but achieved through a different mechanism. This smoothing of the loss landscape leads to better optimization and generalization. A smoother loss landscape implies that the optimization process is less likely to get trapped in poor local minima, and the gradients flow more consistently across the network. This characteristic is particularly beneficial when dealing with complex tasks such as medical image analysis, where the model needs to generalize well from limited data. Hayashi in [22] also illustrates this effect, comparing the loss surfaces of ResNet‐110 without skip connections and DenseNet‐121, showing a significantly smoother landscape for DenseNet.

For MS lesion detection in MRI scans, the comprehensive feature integration capability of DenseNet, in theory, provides significant advantages.

**Figure 8:**
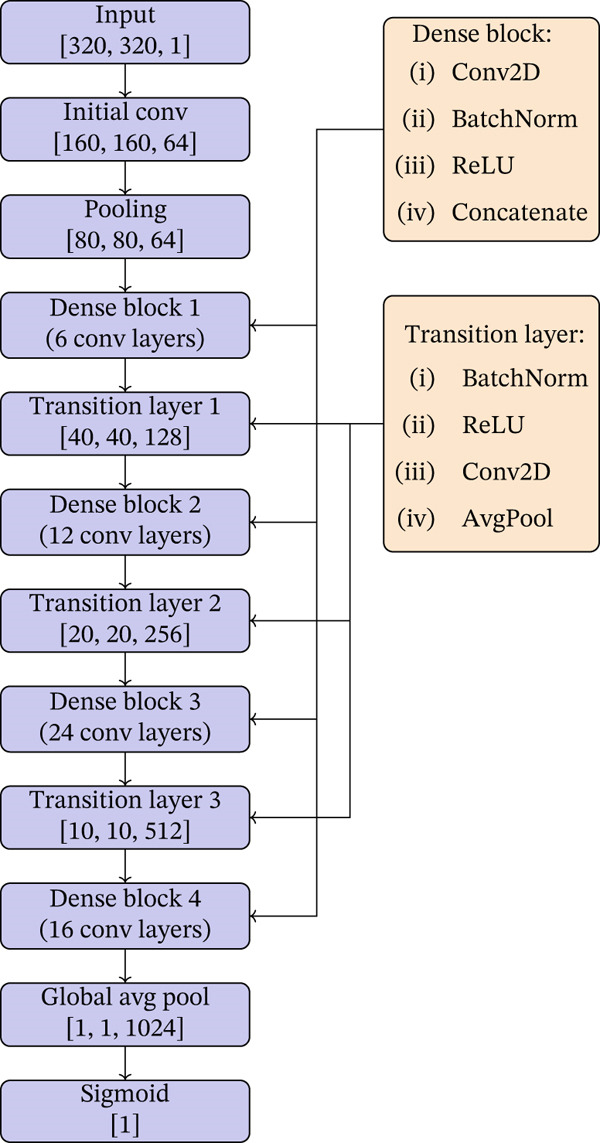
DenseNet architecture for MS lesion detection in MRI.

For MS lesion detection, the DenseNet‐121 architecture illustrated in Figure 8 is used. The architecture is described as follows:

• *Input layer*: Configured to process grayscale images of size 320 × 320 pixels, suitable for the standardized MRI scans used in our dataset.
• *Initial convolutional layer*: Begins with a 7 × 7 convolutional layer with 64 filters, followed by batch normalization and ReLU activation to efficiently capture the preliminary features from the MRI images.
• *Dense blocks*: In the context of DenseNet, a dense block is a group of convolutional layers where each layer receives input from all preceding layers within the same block. Specifically, each block contains layers of 3 × 3 convolutions. The output of each convolutional layer is concatenated with its input, leading to a progressive increase in feature maps while promoting feature reuse. This architecture enhances the flow of information and gradients throughout the network, facilitating more efficient learning.
• *Transition layers*: Incorporated between dense blocks to reduce the dimensionality of the feature maps and to help in compressing the model, making it computationally efficient.
• *Global Average Pooling*: Placed at the end of the last dense block to transform the feature map into a single‐dimensional vector, reducing the total number of parameters and helping to prevent overfitting.
• *Output layer*: Concludes with a dense layer that uses a sigmoid activation function, optimized for binary classification to detect the presence or absence of MS lesions.

The DenseNet model′s ability to preserve the feature integrity throughout the network makes it suitable for medical image analysis, where preserving spatial hierarchies in the features is crucial.

### 3.5. Training

The training of each model—AlexNet‐C, pretrained VGG16, ResNet‐10, and DenseNet‐121—was conducted using the Adam optimizer and binary cross‐entropy loss function. The models were evaluated using accuracy, precision, recall, and *F*1‐score metrics.

The optimal learning rate for each model was determined through a random grid search to ensure effective convergence and performance. The learning rates for each model are shown in Table 1.

**Table 1:** Learning rates determined through random grid search for each model.

Model	Learning rate
AlexNet‐C	5 × 10^−4^
Pretrained VGG16	5 × 10^−6^
ResNet‐10	5 × 10^−5^
DenseNet‐121	5 × 10^−5^

The consistent use of the Adam optimizer and binary cross‐entropy loss function across all models provided a stable training environment, facilitating effective optimization and convergence.

## 4. Results

This section presents the performance metrics obtained from the evaluation of the different models used in this study for MS detection. The key metrics reported include precision, recall, *F*1‐score, accuracy, confusion matrix components (true positives [TPs], true negatives [TNs], false positives [FPs], and false negatives [FNs]), receiver operating characteristic (ROC) curves, and AUC values.

### 4.1. Model Complexity

The complexity of each model, reflected by its size and inference time, is summarized in Table 2. The size of a model can impact its performance, training time, and the amount of computational resources required in both training and inference modes.

**Table 2:** Model sizes and inference times reflecting model complexity.

Model	Size (MB)	Inference time (ms)
AlexNet‐C	810.8	16
Pretrained VGG16	116.3	80
ResNet‐10	3.7	27
DenseNet‐121	113.8	103

### 4.2. ROC Curves and AUC Analysis

**Figure 9:**
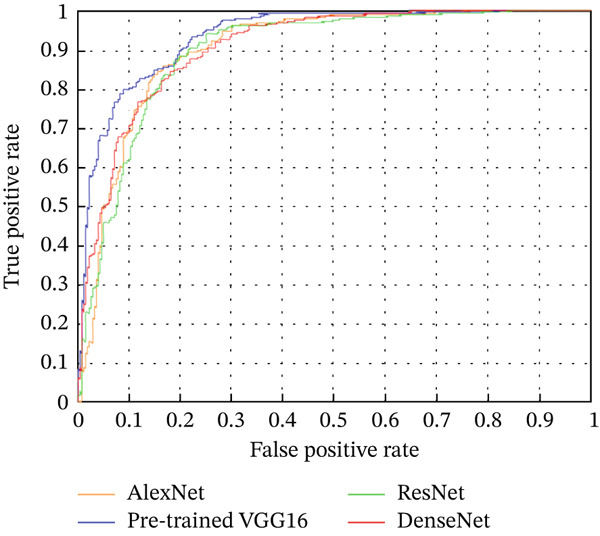
ROC curves for different models.

Figure 9 presents the ROC curves for all the models, which illustrate the trade‐off between the true‐positive rate (TPR) and the false‐positive rate (FPR). The curves indicate the discriminative ability of each model. Among the models, pretrained VGG16 shows the highest curve, suggesting better performance in distinguishing between the positive and negative classes.

In addition to the ROC curves, Table 3 reports the AUC values for each model. The pretrained VGG16 model achieved the highest AUC (0.94), indicating superior overall performance in classification tasks. DenseNet‐121 followed with an AUC of 0.91, while AlexNet‐C and ResNet‐10 both achieved an AUC of 0.90.

**Table 3:** Models performances in terms of AUC.

Model	AUC
AlexNet‐C	0.90
Pretrained VGG16	0.94
ResNet‐10	0.90
DenseNet‐121	0.91

The threshold of prediction was optimized based on the ROC curve by choosing the threshold that maximizes TPR‐FPR, known as Youden′s *J* statistic. The optimal threshold is calculated as follows:

J=TPR−FPR,Optimal threshold=argmaxthresholdJ.

### 4.3. Confusion Matrix Analysis

Table 4 provides a detailed comparison of the confusion matrix components—TN, FP, FN, and TP—for each model. The pretrained VGG16 model achieved the highest rate of TP at 46.19% and the lowest rate of FN at 3.53%. ResNet‐10 also performed well, with a high rate of TP (46.56%) and a comparable rate of FN (3.17%).

**Table 4:** Comparison of confusion matrices for different models.

Model	TP (%)	FP (%)	TN (%)	FN (%)
AlexNet‐C	41.96	7.94	42.35	7.76
Pretrained VGG16	46.19	11.11	39.16	3.53
ResNet‐10	46.56	12.52	37.74	3.17
DenseNet‐121	41.81	9.35	40.91	7.94

### 4.4. Classification Metrics

The classification performance of each model is summarized in Table 5. The metrics reported include precision, recall, *F*1‐score, and accuracy. Among the models, pretrained VGG16 achieved the highest accuracy (0.85), followed closely by ResNet‐10 and AlexNet‐C, both at 0.84. The DenseNet‐121 model showed slightly lower performance across these metrics.

**Table 5:** Summary of classification reports for different models.

Model	Precision	Recall	*F*1‐score	Accuracy
AlexNet‐C	0.84	0.84	0.84	0.84
Pretrained VGG16	0.81	0.93	0.86	0.85
ResNet‐10	0.79	0.94	0.86	0.84
DenseNet‐121	0.82	0.84	0.83	0.83

### 4.5. External Validation on an Independent Dataset

To evaluate generalization beyond the original dataset, the selected ResNet‐10 model was validated on an independent MRI dataset previously used in our earlier work on brain inflammatory disease detection (Table 6) [23]. Slices were manually labeled into MS and non‐MS, and evaluation was performed on a balanced subset by randomly selecting an equal number of MS and non‐MS slices. The decision threshold was kept fixed using the previously selected operating point (optimized on the original validation set) to avoid tuning on the external dataset.

**Table 6:** External validation performance of the ResNet‐10 on an independent dataset (balanced evaluation with equal MS and non‐MS slices). Metrics shown are for MS detection (positive class).

Precision	Recall	*F*1‐score	Accuracy
0.75	0.98	0.85	0.83

### 4.6. Model Interpretability Using Gradient‐Weighted Class Activation Mapping (Grad‐CAM)

To qualitatively inspect the decision‐making of the selected ResNet‐10 model, Grad‐CAM was applied to generate saliency heatmaps highlighting the image regions that most influenced the MS prediction. Figure 10 shows a representative example where the Grad‐CAM response strongly overlaps lesion areas while also activating broader anatomical patterns that correlate with slice location and typical MS lesion distribution.

Figure 10Qualitative interpretability example. Grad‐CAM highlights lesion‐relevant regions and additional anatomical/texture cues that correlate with brain slice location and regions where MS lesions are more probable.

**Figure:**
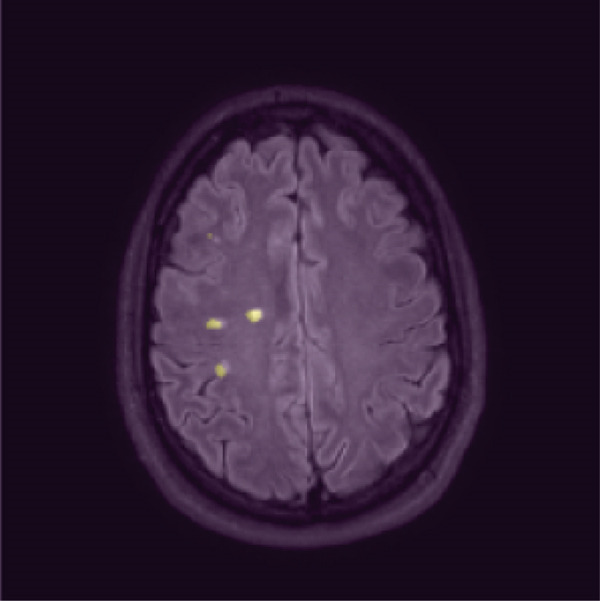
(a) Lesion mask overlay (reference annotation)

**Figure:**
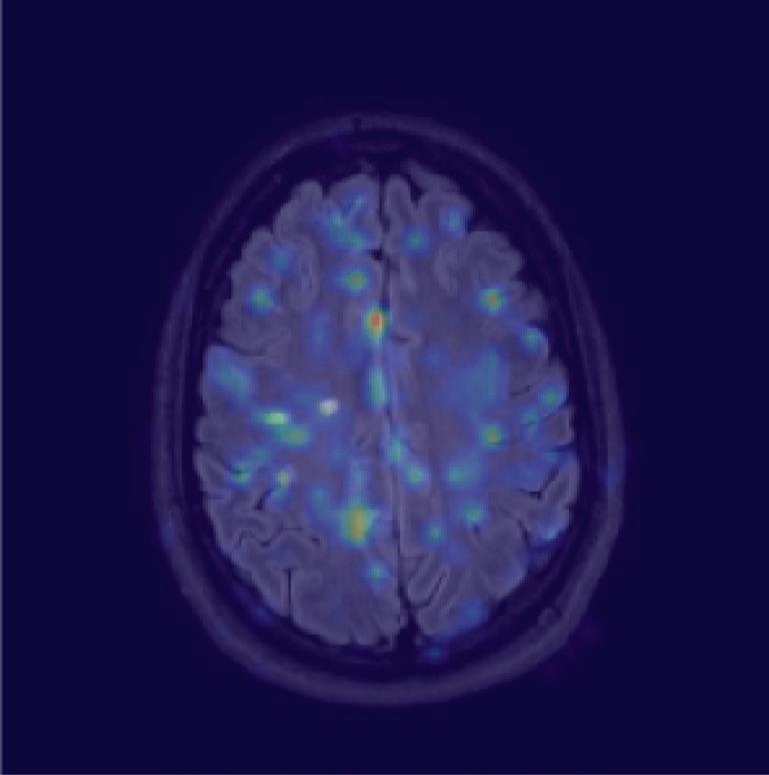
(b) Grad‐CAM overlay (model attention)

## 5. Discussion

### 5.1. Model Performance Comparison

1. *Accuracy and general performance*: Among the models, the pretrained VGG16 achieved the highest accuracy at 0.85, indicating its superior effectiveness in correctly classifying MS lesions. This result is consistent with its AUC value of 0.94, further demonstrating its strong performance. The robust results from VGG16 can be attributed to transfer learning, which leverages the features learned from the extensive ImageNet dataset, providing a rich feature set fine‐tuned for MS lesion detection.

ResNet‐10 and AlexNet‐C followed closely with accuracies of 0.84. ResNet‐10′s architecture, characterized by its use of skip connections, likely contributed to its success by maintaining gradient flow and preventing the vanishing gradient problem, which is critical for deep feature learning. AlexNet‐C also performed well, particularly due to its regularization techniques such as dropout and L2 regularization, which effectively managed overfitting—a common challenge in medical imaging due to smaller datasets.

DenseNet‐121, while still performing well with an accuracy of 0.83, lagged slightly behind the other models. Despite its dense connectivity, which theoretically enhances feature reuse and gradient flow, the higher complexity of DenseNet‐121 may not have been fully advantageous for this particular task, especially given the relatively small dataset size.

2. *Precision, recall, and* *F*1*-score*: AlexNet‐C excelled in precision with a score of 0.84, effectively minimizing FPs—a critical factor in ensuring that healthy tissues are not misclassified as lesions. However, the pretrained VGG16 and ResNet‐10 models outperformed in recall, with scores of 0.93 and 0.94, respectively. High recall is crucial in medical diagnostics to ensure that most MS lesions are detected.

The *F*1‐scores, which balance precision and recall, were highest for VGG16 and ResNet‐10, both achieving 0.86. This indicates their effectiveness in striking a good balance between detecting TPs and avoiding FPs.

3. *ROC and AUC analysis*: The ROC curves and AUC values further illuminate each model′s ability to distinguish between classes across different threshold settings. VGG16 led the pack with an AUC of 0.94, reinforcing its superior performance. DenseNet‐121, with an AUC of 0.91, slightly outperformed AlexNet‐C and ResNet‐10, both of which had an AUC of 0.90. Despite DenseNet‐121’s lower overall metrics, its higher AUC suggests it possesses strong discriminative power, potentially benefiting from a better threshold optimization strategy focused on maximizing the *F*1‐score. However, the minimal difference in AUC between DenseNet‐121 and the other models (0.01) indicates that, in terms of discriminative power, AlexNet‐C, ResNet‐10, and DenseNet‐121 are quite comparable.
4. *Confusion matrix analysis:* The confusion matrix analysis provides further insights into the errors made by each model. The pretrained VGG16 achieved the highest TP rate (46.19%) and the lowest FN rate (3.53%), demonstrating its effectiveness in accurately identifying MS lesions. ResNet‐10 also performed well, with a TP rate of 46.56% and a FN rate of 3.17%, highlighting its strong detection capabilities.

### 5.2. Model Complexity and Suitability

The complexity of each model, particularly in terms of size and inference time, is a critical factor in determining its suitability for deployment, especially in resource‐constrained environments.

ResNet‐10, despite its significantly smaller size (3.7 MB) compared to AlexNet‐C (810.8 MB) and VGG16 (116.3 MB), achieved comparable performance. This efficiency aligns with the principle of Occam′s razor, which favors simpler models that can explain the data adequately. However, it takes 68% longer than AlexNet‐C and 66% faster than VGG16 in inference mode.

Although DenseNet‐121 demonstrated a slightly higher AUC than ResNet‐10 and AlexNet‐C, its overall performance metrics were lower, and its complexity was significantly higher. This suggests that while DenseNet‐121 is powerful, it may be unnecessarily complex for the task at hand, particularly when a simpler model like ResNet‐10 can achieve similar or better performance with much lower complexity.

In conclusion, while all models demonstrated strong performance, the pretrained VGG16 stands out for its overall accuracy and robust AUC, making it a top candidate for MS lesion detection. However, ResNet‐10 offers a compelling balance of performance and model efficiency, making it a highly suitable choice, particularly in scenarios where computational resources are limited. Conversely, when execution speed is the primary concern, regardless of model size, AlexNet‐C demonstrates the fastest inference time while delivering performance comparable to ResNet‐10.

### 5.3. Grad‐CAM Interpretation

The Grad‐CAM visualization for the ResNet‐10 model suggests that the model relies on two complementary signal types. First, high‐response regions overlap with lesion‐like hyperintensities, consistent with lesion‐driven decision‐making. Second, the activation is not limited to lesion pixels and also appears on broader anatomical/texture patterns that encode where the slice lies within the brain. This indicates that the model implicitly learns location‐dependent priors from training data (i.e., which anatomical regions and slice levels are more likely to contain MS lesions) in addition to detecting lesion appearance itself. This combination is coherent with clinical reality, where lesion probability is not uniform across brain regions, and supports the model′s ability to integrate local pathological evidence with global contextual cues.

### 5.4. Generalization on Independent Data

External validation of the ResNet‐10 model on an independent dataset from prior work [23] demonstrates that the model′s learned representation transfers beyond the dataset used for training and internal testing. Using a balanced evaluation design and a fixed decision threshold, the model achieved 0.833 accuracy with high MS recall (0.98) and an *F*1‐score of 0.85. These results support the model′s robustness to dataset shift and indicate that performance is not limited to a single data source.

## 6. Conclusion and Future Work

In this study, we evaluated the performance of four CNN architectures—AlexNet‐C, pretrained VGG16, ResNet‐10, and DenseNet‐121—for the task of MS lesion detection in MRI images. The pretrained VGG16 model demonstrated the highest accuracy and AUC, highlighting its effectiveness in classifying MS lesions. ResNet‐10, despite its smaller size, offered a compelling balance of performance and efficiency, making it a suitable option for deployment in resource‐constrained environments.

This study faced several limitations, including the relatively small dataset available, which may have restricted the model′s ability to generalize to unseen data, although this dataset was the only freely and openly accessible resource for similar studies. The experiments were also constrained by the limited computational resources, which may have hindered the exploration of more complex models or larger datasets. Additionally, the robustness and generalizability of the models to other datasets remain uncertain, as the study was conducted on a single dataset. Finally, the “black box” nature of the deep learning models used poses challenges in interpreting their decision‐making processes, which could impede clinical acceptance.

Future work to address these limitations and enhance the models could focus on several key areas: expanding the dataset by incorporating more MRI images from diverse sources to improve generalization and robustness; exploring advanced preprocessing techniques, such as sophisticated image denoising and normalization, to enhance input data quality and model performance; leveraging transfer learning from pretrained models on larger and more diverse medical imaging datasets or unrelated MRI tasks to improve feature extraction and generalizability; investigating self‐supervised learning methods, such as autoencoders and image‐based joint embedding predictive architectures (I‐JEPA), to boost feature extraction and model robustness without extensive labeled data; improving model interpretability through techniques like rule extraction and the use of semantic features to facilitate clinical acceptance and trust; and integrating postprocessing techniques, such as lesion refinement and 3D volumetric analysis, to enhance the accuracy and clinical relevance of MS lesion detection systems.

## Funding

No funding was received for this manuscript.

## Ethics Statement

This article does not contain any studies with human participants performed by any of the authors.

## Conflicts of Interest

The authors declare no conflicts of interest.

## Data Availability

The data that support the findings of this study are available from the corresponding author upon reasonable request.
